# Impact of exposure of particulate matters on stroke risk: exploring the influence of physical activity among middle-aged and older adults in China

**DOI:** 10.3389/fpubh.2025.1595748

**Published:** 2025-06-12

**Authors:** Zhongning Fang, Pengwei Hou, Chenzhu Cai, Xieli Guo, Mingfa Cai

**Affiliations:** Department of Neurosurgery, Shanghai Sixth People's Hospital Fujian, Jinjiang, China

**Keywords:** particulate matters, stroke, physical activity, China, middle-aged and older adults

## Abstract

**Background:**

Particulate matter is increasingly recognized as a critical environmental risk factor for stroke, particularly among older populations. Although physical activity confers substantial cerebrovascular benefits, it remains unclear how it might mediate or moderate the adverse influence of different sizes of particulate matter on stroke risk.

**Methods:**

A prospective cohort analysis was conducted using data from the China Health and Retirement Longitudinal Study, which enrolled adults aged ≥45 years from diverse regions across mainland China. Annual mean concentrations of PM_1_, PM_2.5_, and PM_10_ were estimated from 2010 to 2020 based on geocoded residential information. Incident stroke cases were identified through self-reported diagnoses and hospitalization records. Binary logistic mixed-effect models examined the associations between exposures to PM_1_, PM_2.5_, and PM_10_, respectively, and stroke risk. Subgroup and mediation analyses explored the roles of physical activity, gender, and job status.

**Results:**

Of 13,573 participants, 540 (4.0%) experienced an incident stroke during follow-up. After full adjustment for covariates, each 10 μg/m3 increment in PM_1_, PM_2.5_, and PM_10_ was significantly associated with higher stroke odds (odds ratios = 1.08, 1.05, and 1.04, all *P* < 0.01). Stronger relationships were observed among physically inactive individuals, women, and those who were unemployed or engaged in agricultural work. Mediation analysis indicated that physical activity accounted for ~19.6% of the detrimental effect of elevated PM_2.5_ on stroke likelihood, suggesting that reduced engagement in physical activity constitutes an important pathway through which finer particulate pollution exerts its harmful impact.

**Conclusions:**

Greater exposure to PM_1_, PM_2.5_, and PM_10_ substantially elevates the risk of stroke among middle-aged and older adults in China, especially in subgroups characterized by lower levels of physical activity and socioeconomic disadvantage. Physical activity partially mediates the effect of PM_2.5_ on stroke risk, underscoring the need for integrated public health interventions that address both environmental pollution and modifiable lifestyle factors. Future studies utilizing high-resolution exposure assessments and objective health measures could further elucidate causal mechanisms and guide strategies to mitigate pollution-related stroke.

## Introduction

Stroke is one of the leading causes of mortality and disability worldwide, creating a substantial burden for healthcare systems and societies (1). According to the Global Burden of Disease Study 2019, the global incidence and prevalence of stroke have persisted at high levels, with low- and middle-income countries, including China, exhibiting particularly rapid increases (2). In China, the stroke burden is exacerbated by a rapidly aging population and widespread exposure to modifiable risk factors such as air pollution and physical inactivity (1, 3).

Numerous epidemiological investigations have implicated air pollution, especially particulate matters, as a key environmental determinant in cardiovascular and cerebrovascular events (4, 5). Fine particulate matter, such as PM_2.5_, can penetrate the alveolar-capillary barrier and enter systemic circulation, triggering oxidative stress, endothelial dysfunction, and inflammatory pathways that collectively heighten stroke risk (5). Indeed, a systematic review and meta-analysis revealed that higher PM_2.5_ levels are significantly associated with both ischemic and hemorrhagic stroke incidence (6). Although national regulations have contributed to declining air pollutant concentrations in some urban centers, large segments of the population remain exposed to levels surpassing recommended thresholds (7).

Beyond environmental determinants, behavioral factors also influence cerebrovascular outcomes. Physical activity, in particular, has been broadly recognized as a protective factor against stroke and other chronic conditions through its beneficial effects on blood pressure, glucose metabolism, and vascular integrity (8). However, it remains unclear to what extent physical activity interacts with or mediates the adverse consequences of particulate matter on stroke risk in populations with elevated pollution exposure. Notably, older individuals may be disproportionately susceptible to air pollution, while also tending to engage less frequently in moderate or vigorous physical activity (6, 9). Understanding the interplay between particulate matter and physical activity in shaping stroke risk is therefore essential for developing comprehensive preventive strategies.

Against this backdrop, the present study aimed to investigate the impact of PM_1_, PM_2.5_, and PM_10_ exposures on the risk of stroke in a large, nationally representative sample of middle-aged and older Chinese adults, and to clarify whether or how physical activity mediates or modifies this relationship. By elucidating the mechanisms and pathways that link particulate matter exposure to stroke risk, our findings may help inform targeted public health interventions and policy initiatives aimed at mitigating pollution hazards and promoting active lifestyles in vulnerable populations.

## Methodology

### Study design and population

This study employed a prospective cohort framework using data from the China Health and Retirement Longitudinal Study (CHARLS), a nationally representative survey of Chinese adults aged 45 and above conducted by Peking University. Initiated in 2011, CHARLS has continued with biennial follow-up assessments (2013, 2015, 2018, and 2020), utilizing a stratified, multi-stage sampling procedure to achieve broad coverage of diverse regions and population groups across mainland China. Individuals were eligible if they were at least 45 years old at baseline, had no self-reported history of stroke, and provided valid residential information for geocoding. Participants lacking essential variables, including data on physical activity or stroke outcomes, were excluded to maintain data integrity. The study received ethical approval from the Institutional Review Board of Peking University (IRB00001052-11015), and written informed consent was obtained from all participants.

**Figure 1:**
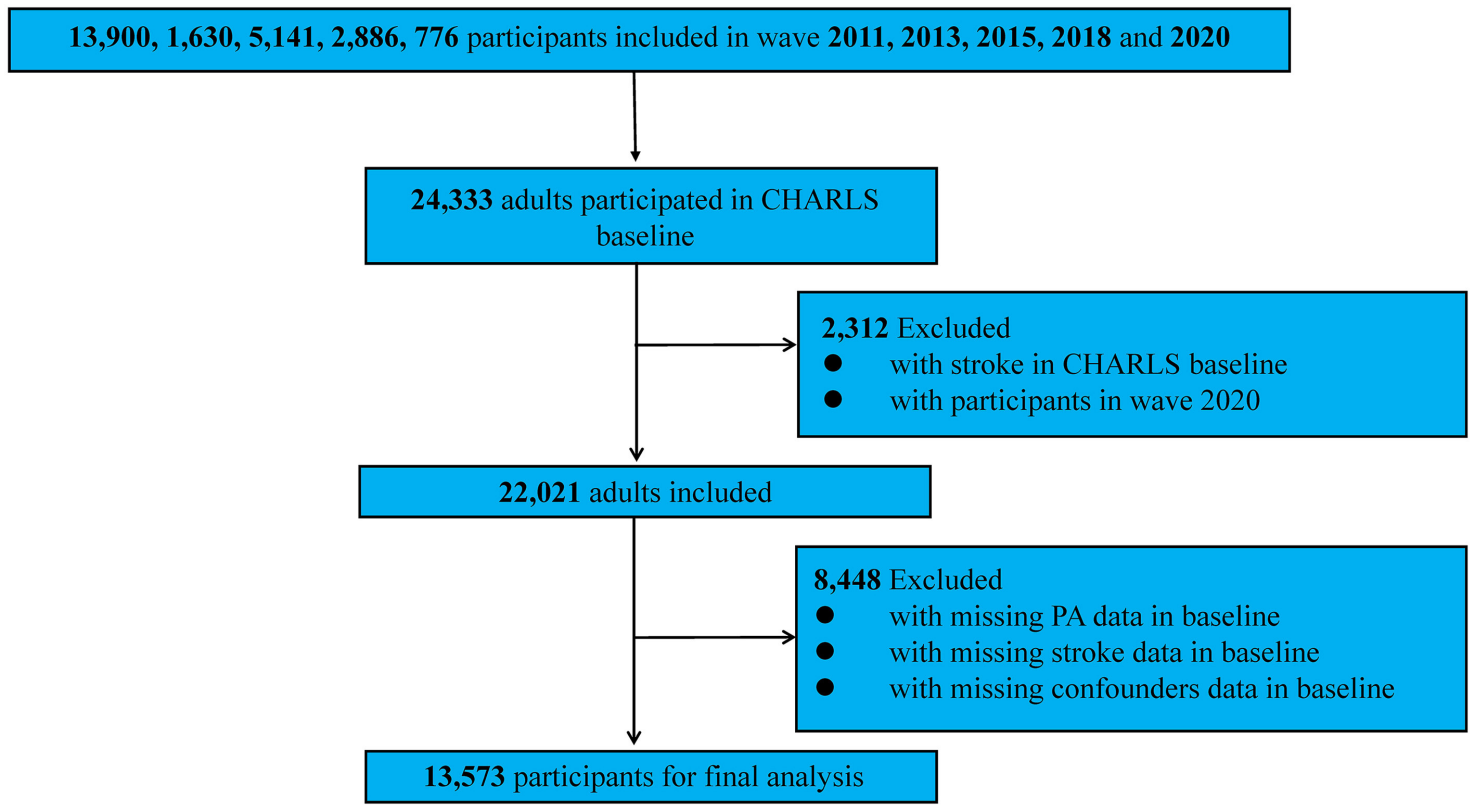
The detailed screening process of selecting the participants of the study for the impact of exposure of particulate matters on stroke risk. PA, physical activity.

Participants were tracked via unique identifiers across five survey waves in 2011, 2013, 2015, 2018, and 2020. Wave 2011 included 13,900 respondents, wave 2013 had 1,630, wave 2015 involved 5,141, wave 2018 encompassed 2,886, and wave 2020 included 776, giving a combined baseline total of 24,333 individuals. Of these, 2,312 people who were stroke at baseline and everyone in the 2020 wave (owing to no subsequent follow-up) were excluded, along with participants missing stroke data (*n* = 3,566), physical activity information (*n* = 435), or covariates (*n* = 4,447). The final analytic sample therefore comprised 13,573 participants (Figure 1).

### Exposures of particulate matters

Annual mean concentrations of PM1, PM_2.5_, and PM10 were obtained from the China High Air Pollutants database, which merges ground-based station data, satellite remote sensing, and sophisticated modeling to deliver pollution estimates at a fine spatial resolution (1 km × 1 km) for the entire country (10, 11). Specifically, PM_1_, PM_2.5_, and PM_10_ measures spanning January 2010 to December 2020 were extracted to match each participant's residential coordinates. Exposure assignments were based on pollutant levels from the previous 6 months to account for lag effects; updated addresses were used to recalculate values if individuals relocated between waves. These estimates, recorded in micrograms per cubic meter (μg/m3), served as the principal independent variables in the subsequent analyses.

### Stroke evaluation

The primary outcome was incident stroke, identified at any post-baseline wave through self-reported medical diagnoses or, whenever available, hospitalization and physician records. Individuals who already had a stroke diagnosis at baseline were excluded to ensure that only newly developed events were counted. Time-to-event was anchored at the date of the baseline interview (2011) and extended until the initial stroke diagnosis, attrition from the cohort, or culmination of the final wave in 2020, whichever came first.

### Covariates

Building on insights from previous investigations (12–14), a range of confounders was incorporated into the analysis. Specifically, (1) demographic factors consisted of age group (<70, 70–80, or ≥80 years), gender (male or female), educational level (illiterate, primary school and below, middle school and high school, technical school and above), residence (urban or rural), and marital status (married or unmarried). (2) Health-related behaviors included smoking status (still smoking, former smoking, or never smoking), alcohol consumption (still drinking, former drinking, or never drinking), sleep duration (short <7 h, normal 7–9 h, or long >9 h), physical activity (physical inactivity or physically active) and BMI group (Underweight, 15 kg/m^2^ < BMI < 18.5 kg/m^2^; Normal weight, 18.5 kg/m^2^ ≤ BMI < 24 kg/m^2^; Overweight, 24 kg/m^2^ ≤ BMI < 28 kg/m^2^; or Obesity, 28 kg/m^2^ ≤ BMI < 40 kg/m^2^). (3) Socioeconomic measures encompassed medical insurance [Urban employee medical insurance (UEMI); Urban and rural resident medical insurance (URRMI); Urban resident medical insurance (URMI); New cooperative medical insurance (NCMI)], income group [low income, personal annual income of 0–20,000¥ (~2,779.20$); middle income, personal annual income of 20,000–50,000¥ (~6,947.99$); high income, personal annual income over 50,000¥ (~6,947.99$)], and job status (unemployed, agricultural job, and non-agricultural job).

### Physical activity assessment

The participants' levels of physical activity were assessed using a modified version of the International Physical Activity Questionnaire (IPAQ). During each of five survey waves (2011–2020), individuals reported any physical activity undertaken for at least 10 min, which was categorized into vigorous (carrying heavy loads, digging, farming, aerobics, fast cycling, or cycling with cargo), moderate (moving lighter loads, cycling at a regular pace, mopping, practicing tai chi, or walking at a brisk speed), or light (walking for work-related tasks or leisure). Respondents also indicated both the weekly frequency, ranging from once to seven times, and the duration of these activities, grouped into specified intervals (15, 16).

Because the questionnaire did not capture precise periods, a median-based procedure was employed, consistent with previous studies. Reported intervals were mapped to approximate durations: more than 10 min but <30 min was treated as 20 min, above 30 min but below 2 h as 75 min, 2–4 h as 180 min, and beyond 4 h as 240 min. The total weekly time for each physical activity intensity category was then computed by multiplying the number of days an activity was performed by its corresponding median duration. Next, a metabolic equivalent (MET) index was derived by multiplying the weekly time (minutes) for vigorous-intensity activities by 8.0, moderate-intensity by 4.0, and light-intensity by 3.3. Consistent with IPAQ guidelines, individuals whose overall weekly physical activity volume (MET-minutes per week) was <600 were considered physically inactive (15, 17).

### Statistical analysis

At baseline (wave 2011), participants were characterized by frequencies (*n*) and proportions (%), stratified according to stroke status (stroke-free vs. stroke cases). Because numerous variables were included, the Kolmogorov–Smirnov test was employed to determine whether pollutant concentrations followed a normal distribution. Variables with normal distributions were summarized by mean and standard deviation (SD), whereas skewed variables were expressed as medians alongside interquartile ranges (25th, 75th).

Rather than employing a conventional time-to-event model, we utilized a binary logistic mixed-effects model in this study for several reasons. First, the CHARLS collects data in discrete waves (2011, 2013, 2015, 2018, and 2020), spaced ~2–3 years apart, making the exact time of stroke onset less precisely determined (18). Second, our focus lies in how evolving exposures to particulate matter and time-varying covariates jointly influence stroke odds, rather than modeling the precise time until stroke occurrence. By using a binary logistic mixed-effects approach, we can accommodate the repeated-measures structure, where stroke status (present vs. absent) is recorded in each wave, and include a random intercept for each participant, thus accounting for inter-individual variability and within-subject correlation. In addition, although the mixed-effects models can accommodate unbalanced data, we excluded participants from the 2020 wave because we aimed to focus on stroke risk in a cohort-like framework. CHARLS includes newly enrolled participants at each wave, which effectively serves as a “baseline” for those new enrollees, followed by subsequent waves to assess stroke incidence. Since the 2020 wave is the most recent with no additional follow-up data, it cannot contribute to assessing new stroke events beyond that wave. As a result, individuals whose first participation was in 2020 were excluded in a manner analogous to how baseline years are excluded in time-to-event analyses when no further follow-up is available.

Several models were estimated for each pollutant: Model I was unadjusted, while Model II added demographic factors such as age group and gender. Model III incorporated broader socioeconomic characteristics—education, urban or rural residence, marital status, medical insurance coverage, income quintile, and job status, and an additional factor for survey wave. Model IV further introduced health-related variables, including smoking status, alcohol consumption, sleep duration, physical activity, and BMI group. From these models, odds ratios (ORs) and 95% confidence intervals (CIs) were computed to assess the relationship between particulate matters and stroke. Natural spline functions were also utilized to capture potential non-linear associations between PM_1_, PM_2.5_, PM_10_, and stroke probability (19).

To determine if the associations were non-linear, two binary logistic mixed-effect models were fitted for each pollutant. One specified a linear term, whereas the other used a natural spline term. A likelihood ratio test compared these two specifications; a significant outcome signaled the presence of non-linearity. Subsequently, by conducting an interaction analysis that incorporated all prespecified covariates, we investigated whether any of these factors altered the association between particulate matters and stroke risk. We observed that only gender, job status, and physical activity displayed significant interactions with stroke risk. Consequently, we carried out subgroup analyses to assess the relationship between air pollutants and stroke risk within each of these significantly interacting covariates. We then conducted a mediation analysis to examine whether physical activity contributed to explaining the association between each particulate matters and stroke risk. Mediation analysis was used to decompose the total effect into direct and indirect pathways, with the latter reflecting the mediating role of physical activity. The significance of the mediation effect was determined using bias-corrected bootstrap confidence intervals derived from 5,000 resamples (20).

Finally, a series of sensitivity analyses was conducted to verify the robustness of the primary findings. First, the main models were re-estimated by implementing Last Observation Carried Forward (LOCF) imputation to observe the impact of missing values, allowing for a direct comparison between results obtained from the imputed and non-imputed datasets. Second, alternative lag structures for air pollution exposure were tested by assigning 1- and 2-year lags instead of the primary lag. This procedure helped evaluate whether different temporal assumptions regarding pollutant effects materially changed the results. Finally, physical activity was recategorized into three groups rather than the original binary designation (physically inactive vs. physically active). The aim was to ascertain whether the more granular physical activity classification yielded findings consistent with the main analysis. All statistical analyses and data visualizations were performed in Stata 16.0 (StataCorp, College Station, TX, USA) and R (version 4.3.2). Statistical significance was concluded at a *P*-value < 0.05.

## Results

### Baseline characteristics of participants

Table 1 displays the baseline characteristics of the 13,573 participants, including 13,033 stroke-free individuals and 540 stroke cases. Overall, those who experienced stroke were significantly older (*P* < 0.001): while most participants were under 70 years of age (85.4% in total), a larger proportion of stroke cases fell into the 70–80 (19.8%) and ≥80 (6.9%) brackets compared with those who were stroke-free. Gender distribution also differed (*P* < 0.001), with men accounting for 58.3% of stroke cases vs. 45.3% in the stroke-free group. Educational level varied notably (*P* < 0.001): 46.7% of stroke cases had only primary schooling compared to 37.3% among the stroke-free, and fewer stroke cases had a technical school or higher degree (7.0 vs. 14.6%). Regarding marital status, a higher proportion of stroke cases were unmarried (15.0 vs. 11.5%, *P* = 0.013). More stroke cases resided in rural areas (72.4 vs. 67.6%, *P* = 0.019). In terms of smoking (*P* < 0.001), stroke cases exhibited higher rates of former smoking (20.7%) and continued smoking (35.0%) compared to the stroke-free group. Although current alcohol drinking (31.1 vs. 25.5%, *P* = 0.007) was more frequent among stroke cases, the proportion of never drinkers (62.2%) remained dominant within this subgroup. BMI classifications also showed significant disparities (*P* < 0.001); stroke cases were more likely to be overweight (38.9 vs. 28.4%) or obese (17.8 vs. 8.7%), but less likely to be underweight (3.7 vs. 7.1%). Medical insurance coverage (*P* < 0.001) differed between groups: stroke cases had relatively higher enrollment in URRMI (16.3 vs. 9.2%), whereas UEMI was less common (3.0 vs. 5.7%). With regard to income (*P* < 0.001), fewer stroke cases had no income (39.6 vs. 68.3%), while more fell into low (28.0%) and middle (27.8%) income categories, compared with 14.7 and 15.3% among the stroke-free, respectively. Employment status (*P* < 0.001) also showed contrasts, as a larger fraction of stroke cases held non-agricultural jobs (39.6 vs. 19.5%), while agricultural work was more frequent among stroke-free individuals (51.4 vs. 36.5% in stroke cases). Although no marked differences emerged in sleep duration (*P* = 0.440) with most participants reporting short (<7 h) or normal (7–9 h) sleep, there was a clear divergence in physical activity: the proportion classified as physically inactive was similar (15.2 vs. 15.0%, *P* = 0.900), yet stroke cases accumulated fewer MET-minutes per week on average (6,069.24 vs. 7,555.08, *P* < 0.001). Lastly, stroke cases experienced higher median exposure to PM_1_, PM_2.5_, and PM_10_ (*P* < 0.001 for all).

**Table 1:** Baseline characteristics of participants by stroke-free (*n* = 13,033) vs. stroke-cases (*n* = 540) in the CHARLS.

**Variables**	**Total (*n* = 13,573)**	**Stroke-free (*n* = 13,033)**	**Stroke-cases (*n* = 540)**	***P*-value**
**Age groups, (years)**				
<70	11,593 (85.4)	11,197 (85.9)	396 (73.3)	<0.001
70–80	1,537 (11.3)	1,430 (11.0)	107 (19.8)	
≥80	443 (3.3)	406 (3.1)	37 (6.9)	
**Gender**, ***n*** **(%)**				
Female	7,350 (54.2)	7,125 (54.7)	225 (41.7)	<0.001
Male	6,223 (45.8)	5,908 (45.3)	315 (58.3)	
**Educational level**, ***n*** **(%)**				
Illiterate	3,127 (23.0)	3,025 (23.2)	102 (18.9)	<0.001
Primary school	5,119 (37.7)	4,867 (37.3)	252 (46.7)	
Middle school and high school	3,389 (25.0)	3,241 (24.9)	148 (27.4)	
Technical school or higher	1,938 (14.3)	1,900 (14.6)	38 (7.0)	
**Marriage**, ***n*** **(%)**				
Unmarried	1,580 (11.6)	1,499 (11.5)	81 (15.0)	0.013
Married	11,993 (88.4)	11,534 (88.5)	459 (85.0)	
**Residence**, ***n*** **(%)**				
Rural area	9,201 (67.8)	8,810 (67.6)	391 (72.4)	0.019
Urban area	4,372 (32.2)	4,223 (32.4)	149 (27.6)	
**Smoke**, ***n*** **(%)**				
Never smoking	8,273 (61.0)	8,034 (61.6)	239 (44.3)	<0.001
Former smoking	1,275 (9.4)	1,163 (8.9)	112 (20.7)	
Still smoking	4,025 (29.7)	3,836 (29.4)	189 (35.0)	
**Drink alcohol**, ***n*** **(%)**				
Never drinking	8,908 (65.6)	8,572 (65.8)	336 (62.2)	0.007
Former drinking	1,175 (8.7)	1,139 (8.7)	36 (6.7)	
Still drinking	3,490 (25.7)	3,322 (25.5)	168 (31.1)	
**BMI group**, ***n*** **(%)**				
Underweight	948 (7.0)	928 (7.1)	20 (3.7)	<0.001
Normal weight	7,485 (55.1)	7,271 (55.8)	214 (39.6)	
Overweight	3,906 (28.8)	3,696 (28.4)	210 (38.9)	
Obesity	1,234 (9.1)	1,138 (8.7)	96 (17.8)	
**Medical insurance**, ***n*** **(%)**				
UEMI	756 (5.6)	740 (5.7)	16 (3.0)	<0.001
URRMI	1,288 (9.5)	1,200 (9.2)	88 (16.3)	
URMI	177 (1.3)	169 (1.3)	8 (1.5)	
NCMI	538 (4.0)	517 (4.0)	21 (3.9)	
UEMI	10,259 (75.6)	9,917 (76.1)	342 (63.3)	
URRMI	555 (4.1)	490 (3.8)	65 (12.0)	
**Income group**, ***n*** **(%)**				
Non-income	9,118 (67.2)	8,904 (68.3)	214 (39.6)	<0.001
Low income	2,065 (15.2)	1,914 (14.7)	151 (28.0)	
Middle income	2,150 (15.8)	2,000 (15.3)	150 (27.8)	
High income	240 (1.8)	215 (1.6)	25 (4.6)	
**Job status**, ***n*** **(%)**				
Unemployed	3,931 (29.0)	3,802 (29.2)	129 (23.9)	<0.001
Agricultural job	6,890 (50.8)	6,693 (51.4)	197 (36.5)	
Non-agricultural job	2,752 (20.3)	2,538 (19.5)	214 (39.6)	
**Sleep duration**, ***n*** **(%)**				
Normal (7–9 h)	5,780 (42.6)	5,560 (42.7)	220 (40.7)	0.440
Short (<7 h)	7,096 (52.3)	6,800 (52.2)	296 (54.8)	
Long (>9 h)	697 (5.1)	673 (5.2)	24 (4.4)	
**Physical activity**, ***n*** **(%)**				
Physical inactivity	1,178 (15.0)	1,096 (15.0)	82 (15.2)	0.900
Physically active	6,673 (85.0)	6,215 (85.0)	458 (84.8)	
MET-minutes per week, (minutes)	7,452.00 (7,018.48)	7,555.08 (7,076.08)	6,069.24 (6,031.74)	<0.001
**Particulate matters, (**μ**g/m**3**)**				
PM_1_	25.6 (15.1)	23.7 (13.5)	26.0 (15.2)	<0.001
PM_2.5_	45.2 (28.4)	42.0 (24.3)	45.8 (29.3)	
PM_10_	78.2 (49.6)	76.5 (44.6)	78.7 (50.1)	

UEMI, Urban employee medical insurance; URRMI, Urban and rural resident medical insurance; URMI, Urban resident medical insurance; NCMI, New cooperative medical insurance.

BMI, body mass index (kg/m^2^); Underweight, 15 < BMI < 18.5; Normal weight, 18.5 ≤ BMI < 24; Overweight, 24 ≤ BMI < 28; Obesity, 28 ≤ BMI < 40; Low income, personal annual income of 0 to 20,000¥ (~2,779.20$); Middle income, personal annual income of 20,000¥ to 50,000¥ (~6,947.99$); High income, personal annual income over 50,000¥ (~6,947.99$); Sleep duration: normal night-time sleep duration, 7–9 h; short night-time sleep duration, <7 h; long night-time sleep duration, >9 h.

### Associations between exposure of particulate matters and stroke risk

**Figure 2:**
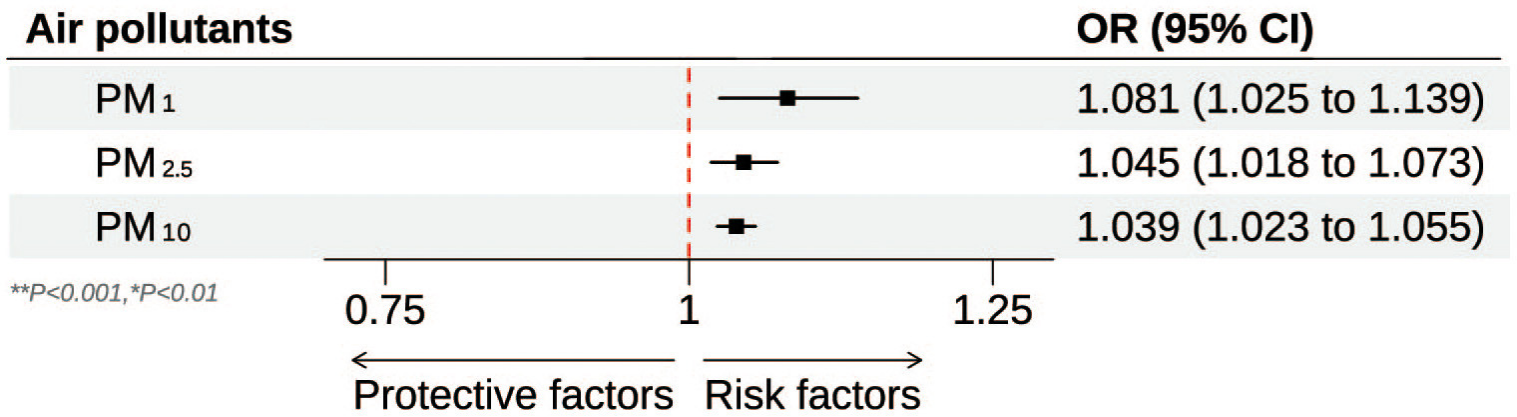
Forest plot of the association between particulate matter (PM_1_^**^, PM_2.5_^**^, and PM_10_^*^) and stroke risk. ^**^P < 0.001; ^*^P < 0.01.

**Figure 3:**
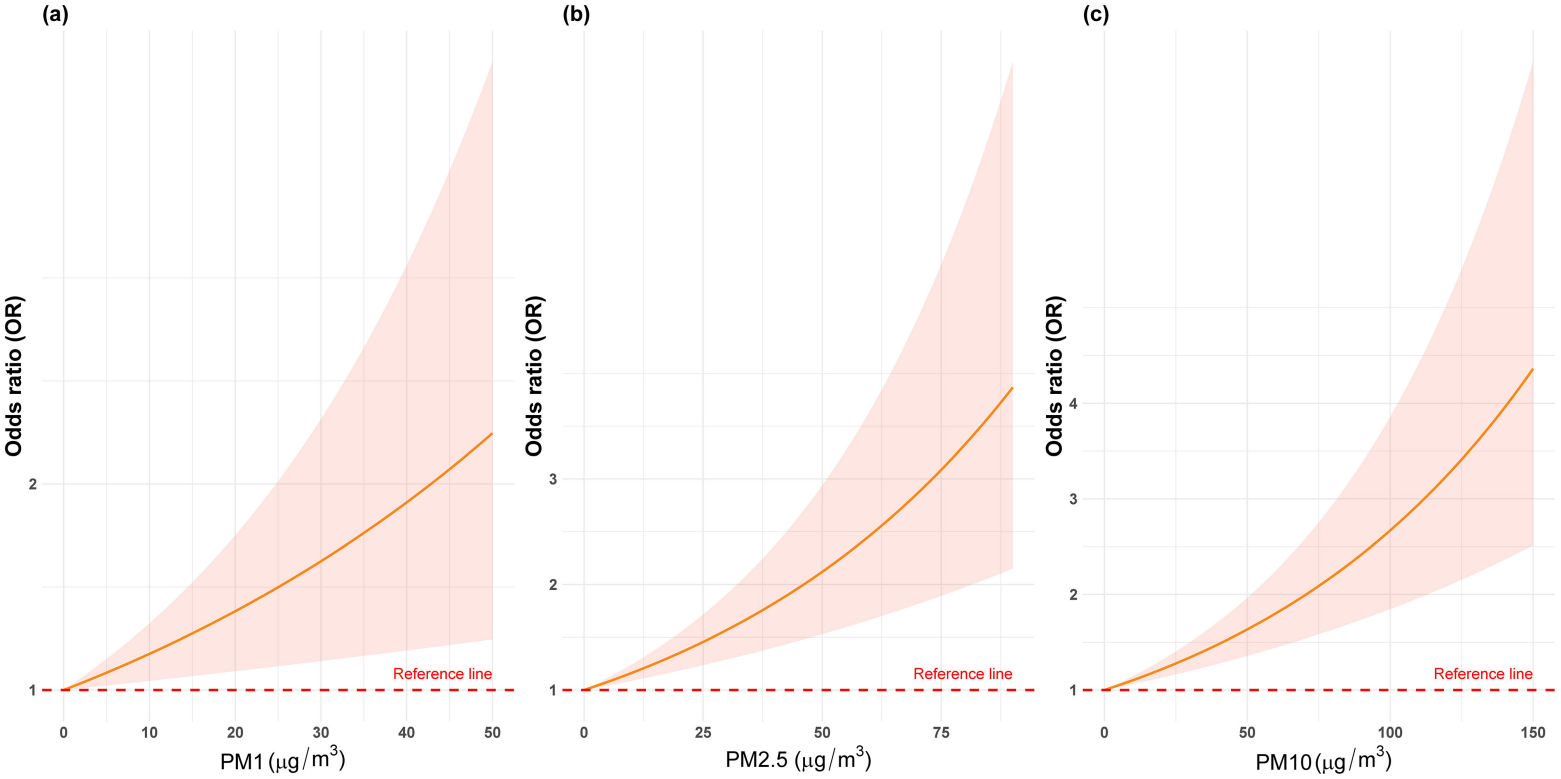
The natural cubic spline analyses of the annual mean concentrations of particulate matters (PM_1_, PM_2.5_, and PM_10_) and stroke risk.

Table 2 and Figure 2 illustrate the progressive adjustment of covariates and their impact on the relationship between particulate matter (PM_1_, PM_2.5_, and PM_10_) and stroke risk. In Model I (no covariates), PM_1_ and PM_2.5_ show weak, potentially protective effects (ORs below or near unity), while PM_10_ demonstrates no association (OR = 1.00, 95% CI: 0.99–1.01). Once age and sex are incorporated in Model II, PM_1_ shifts to an almost neutral effect (OR = 0.99, 95% CI: 0.98–1.00), and both PM_2.5_ and PM_10_ indicate slightly elevated stroke odds. Further inclusion of broader socioeconomic factors in Model III reveals a marked positive association for all three pollutants, most notably PM_1_ (OR = 1.09, 95% CI: 1.04–1.15). After full adjustment in Model IV, each 10 μg/m3 increment in PM_1_, PM_2.5_, and PM_10_ remains significantly linked to stroke (OR = 1.08, 95% CI: 1.03–1.14; OR = 1.05, 95% CI: 1.02–1.07; OR = 1.04, 95% CI: 1.02–1.06, respectively). Figure 3 presents the natural cubic spline analyses of the annual mean concentrations of particulate matter and stroke risk. The *P*-values for non-linearity were 0.001 for PM_1_ and < 0.001 for both PM_2.5_ and PM_10_, indicating that each particulate matter exhibits a linear association with stroke risk. Table 3 displays the outcomes for models incorporating multiple particulate matters. Under single-pollutant conditions, PM_1_, PM_2.5_, and PM_10_ each exhibited a significant positive association with stroke risk in Model IV. Once these three pollutants were introduced simultaneously, the relationships for PM_1_ and PM_2.5_ became slightly attenuated, whereas PM_10_ retained a comparable or mildly elevated effect size. In two-pollutant models (PM_1_ + PM_2.5_ or PM_2.5_ + PM_10_), none of the particulate fractions completely lost significance, indicating that their combined presence partly reflects overlapping pathways yet still yields a collective impact on stroke risk. Moreover, the three-pollutant model produced a modestly higher odds ratio than some two-pollutant configurations, suggesting partial independence in how each fraction contributes to the overall burden.

**Table 2:** The relationship between air pollutants exposure (10 μg/m^3^) and stroke risk in the CHARLS participants.

**Exposures**	**Model I**	**Model II**	**Model III**	**Model IV**
	**OR (95% CI)**	**OR (95% CI)**	**OR (95% CI)**	**OR (95% CI)**
PM_1_	0.95 (0.93–0.96)	0.99 (0.98–1.00)	1.09 (1.04–1.15)	1.08 (1.03–1.14)
PM_2.5_	0.99 (0.98–0.99)	1.01 (1.00–1.02)	1.05 (1.02–1.08)	1.05 (1.02–1.07)
PM_10_	1.00 (0.99–1.01)	1.02 (1.01–1.02)	1.04 (1.03–1.06)	1.04 (1.02–1.06)

Model I, no covariates were adjusted; Model II: adjusted for age group and sex; Model III incorporated broader socioeconomic characteristics-education, urban or rural residence, marital status, medical insurance coverage, income quintile, and job status, and an additional factor for survey wave; Model IV further introduced health-related variables, including smoking status, alcohol consumption, sleep duration, physical activity and BMI group.

**Table 3:** The multiple particulate matters model analysis for the relationship between air pollutants exposure (10 μg/m^3^) and stroke risk.

**Exposures**	**Model I**	**Model II**	**Model III**	**Model IV**
	**OR (95% CI)**	**OR (95% CI)**	**OR (95% CI)**	**OR (95% CI)**
PM_1_	0.95 (0.93–0.96)	0.99 (0.98–1.00)	1.09 (1.04–1.15)	1.08 (1.03–1.14)
PM_2.5_	0.99 (0.98–0.99)	1.01 (1.00–1.02)	1.05 (1.02–1.08)	1.05 (1.02–1.07)
PM_10_	1.00 (0.99–1.01)	1.02 (1.01–1.02)	1.04 (1.03–1.06)	1.04 (1.02–1.06)
PM_1_ + PM_2.5_	1.04 (1.02–1.06)	1.08 (1.05–1.10)	1.12 (1.07–1.17)	1.10 (1.05–1.15)
PM_2.5_ + PM_10_	1.02 (1.00–1.04)	1.05 (1.03–1.07)	1.09 (1.04–1.13)	1.07 (1.03–1.10)
PM_1_ + PM_2.5_ + PM_10_	1.05 (1.02–1.07)	1.09 (1.05–1.11)	1.13 (1.08–1.18)	1.11 (1.06–1.15)

Model I, no covariates were adjusted; Model II: adjusted for age group and sex; Model III incorporated broader socioeconomic characteristics-education, urban or rural residence, marital status, medical insurance coverage, income quintile, and job status, and an additional factor for survey wave; Model IV further introduced health-related variables, including smoking status, alcohol consumption, sleep duration, physical activity and BMI group.

### Subgroup analyses by physical activity, gender, and job status

Table 4 summarizes the stratified relationships between particulate matter exposure and stroke risk according to physical activity status, gender, and job type. For physically inactive individuals, all three pollutants consistently exhibit stronger associations with stroke across increasingly adjusted models: ORs for PM_1_, PM_2.5_, and PM_10_ reach 1.11 (1.04–1.18), 1.08 (1.03–1.13), and 1.07 (1.02–1.13) in Model IV, respectively. Conversely, in the physically active subgroup, none of the pollutants shows a statistically significant relationship once adjusting for all covariates (Model IV). With regard to gender, female participants demonstrate significant positive associations for all three pollutants in fully adjusted models—PM_1_ (OR = 1.08, 95% CI: 1.03–1.14), PM_2.5_ (OR = 1.05, 95% CI: 1.02–1.08), and PM_10_ (OR = 1.05, 95% CI: 1.03–1.06)—while corresponding estimates in men either hover around 1.00 or fail to reach statistical significance. A similar pattern emerges among job-status groups: those who are unemployed or engaged in agricultural work exhibit significant increases in stroke odds with each 10 μg/m3 rise in PM_1_, PM_2.5_, and PM_10_. In the Model IV, unemployed participants show ORs of 1.13 (1.06–1.20) for PM_1_, 1.11 (1.04–1.18) for PM_2.5_, and 1.09 (1.04–1.15) for PM_10_. By contrast, non-agricultural workers display attenuated or non-significant ORs across all pollutants.

**Table 4:** Subgroup analysis of the relationship between air pollutants exposure (10 μg/m^3^) and stroke risk in CHARLS participants by physical activity, gender and job status.

**Exposures**	**Model I**	**Model II**	**Model III**	**Model IV**
	**OR (95% CI)**	**OR (95% CI)**	**OR (95% CI)**	**OR (95% CI)**
**Physical activity**				
**Physical inactive**				
PM_1_	1.05 (1.01–1.10)	1.08 (1.02–1.14)	1.12 (1.05–1.19)	1.11 (1.04–1.18)
PM_2.5_	1.04 (1.01–1.07)	1.07 (1.02–1.12)	1.09 (1.04–1.14)	1.08 (1.03–1.13)
PM_10_	1.02 (0.99–1.05)	1.06 (1.01–1.11)	1.08 (1.03–1.14)	1.07 (1.02–1.13)
**Physical active**				
PM_1_	1.00 (0.97–1.03)	1.02 (0.98–1.06)	1.02 (0.97–1.08)	1.01 (0.96–1.07)
PM_2.5_	0.99 (0.96–1.02)	1.01 (0.97–1.05)	1.03 (0.98–1.07)	1.02 (0.97–1.06)
PM_10_	0.98 (0.96–1.01)	1.00 (0.97–1.03)	1.01 (0.97–1.06)	1.00 (0.96–1.05)
**Gender**				
**Male**				
PM_1_	0.98 (0.93–1.04)	1.00 (0.98–1.03)	1.02 (0.99–1.04)	1.01 (0.99–1.04)
PM_2.5_	1.00 (0.99–1.01)	1.01 (1.00–1.03)	1.03 (0.99–1.07)	1.02 (0.99–1.06)
PM_10_	1.00 (1.00–1.01)	1.01 (1.00–1.02)	1.02 (1.00–1.04)	1.01 (0.99–1.04)
**Female**				
PM_1_	0.97 (0.95–0.98)	1.02 (1.00–1.03)	1.09 (1.04–1.15)	1.08 (1.03–1.14)
PM_2.5_	0.99 (0.98–1.00)	1.02 (1.01–1.03)	1.06 (1.03–1.08)	1.05 (1.02–1.08)
PM_10_	1.00 (1.00–1.01)	1.02 (1.01–1.03)	1.05 (1.03–1.07)	1.05 (1.03–1.06)
**Job status**				
**Unemployment**				
PM_1_	1.06 (1.02–1.10)	1.10 (1.04–1.16)	1.14 (1.07–1.21)	1.13 (1.06–1.20)
PM_2.5_	1.05 (1.01–1.09)	1.09 (1.03–1.14)	1.12 (1.06–1.19)	1.11 (1.04–1.18)
PM_10_	1.04 (1.01–1.07)	1.08 (1.03–1.12)	1.11 (1.05–1.17)	1.09 (1.04–1.15)
**Agricultural job**				
PM_1_	1.03 (1.00–1.06)	1.06 (1.02–1.10)	1.09 (1.04–1.15)	1.08 (1.03–1.14)
PM_2.5_	1.02 (0.99–1.05)	1.05 (1.01–1.09)	1.08 (1.03–1.13)	1.07 (1.02–1.12)
PM_10_	1.01 (0.98–1.03)	1.04 (1.00–1.07)	1.07 (1.02–1.12)	1.06 (1.01–1.11)
**Non-agricultural job**				
PM_1_	1.01 (0.98–1.04)	1.03 (0.99–1.06)	1.05 (1.00–1.10)	1.04 (0.99–1.09)
PM_2.5_	1.00 (0.97–1.03)	1.02 (0.98–1.06)	1.03 (0.98–1.09)	1.02 (0.97–1.07)
PM_10_	0.99 (0.97–1.01)	1.01 (0.98–1.04)	1.02 (0.98–1.07)	1.01 (0.97–1.06)

Model I, no covariates were adjusted; Model II, adjusted for age group and sex; Model III incorporated broader socioeconomic characteristics-education, urban or rural residence, marital status, medical insurance coverage, income quintile, and job status, and an additional factor for survey wave; Model IV further introduced health-related variables, including smoking status, alcohol consumption, sleep duration, physical activity and BMI group.

### Mediation analysis of particulate matters, physical activity, and stroke risk

**Figure 4:**
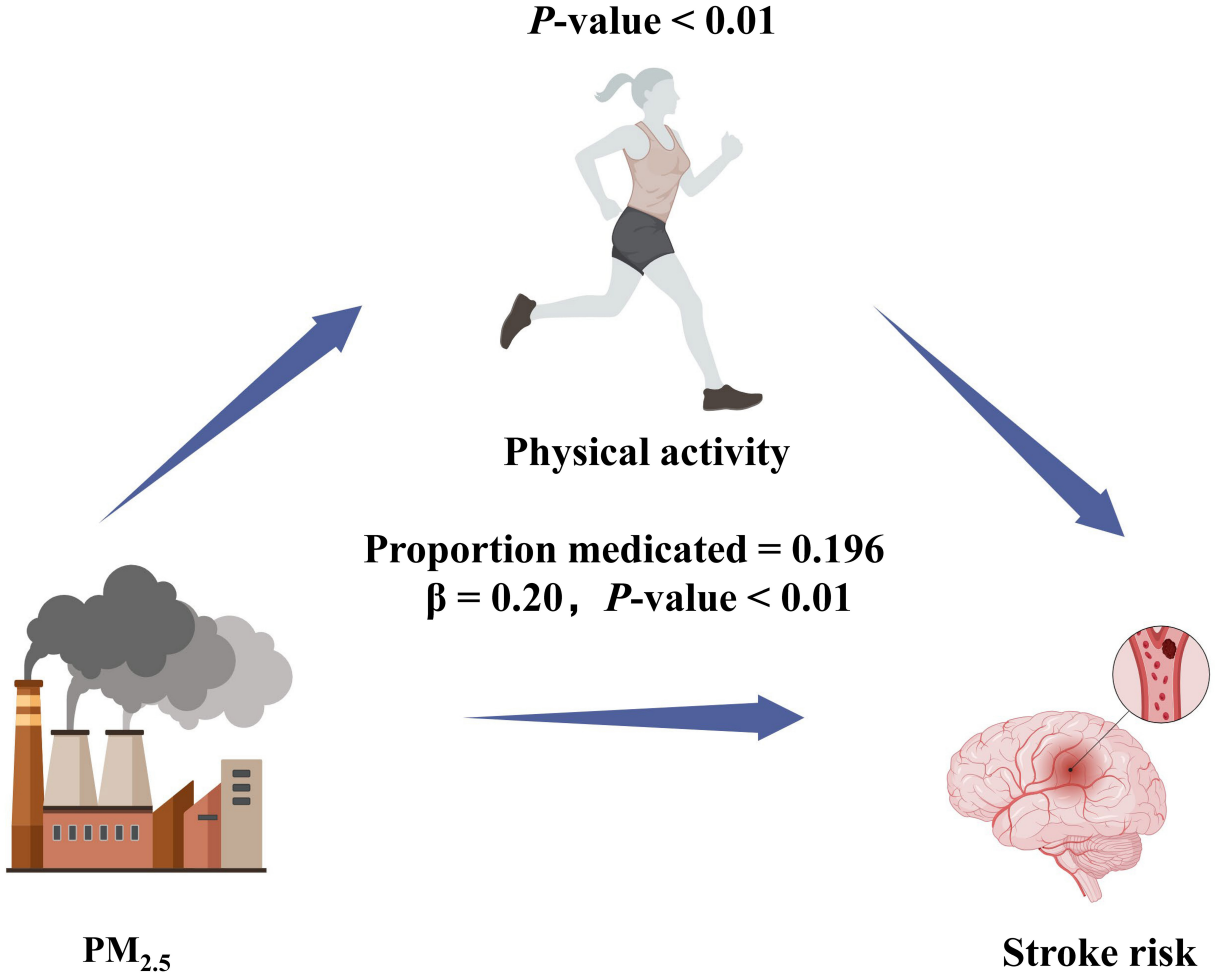
Path diagram of mediation analysis of the relationship between PM_2.5_, physical activity, and stroke risk.

A mediation analysis was performed to determine whether physical activity mediated the relationship between exposure of particulate matters and stroke risk, with each particulate matters as the predictor, physical activity as the mediator, and stroke as the outcome. As illustrated in Figure 4, physical activity accounted for 19.6% of the total effect of higher PM_2.5_ on stroke risk (indirect effect: β = 0.196, *P* < 0.01), suggesting that part of the harmful impact of PM_2.5_ on stroke risk was attributable to lower levels of physical activity. By contrast, no significant mediation effect was found for PM_1_ or PM_10_: the indirect effects of these pollutants on stroke via physical activity (β = 0.05, *P* = 0.44 for PM_1_; β = 0.03, *P* = 0.37 for PM_10_) were not statistically significant.

### Sensitivity analyses

To assess the robustness of our primary findings, a series of sensitivity analyses was undertaken. First, we used LOCF imputation to address missing data and compared results derived from the imputed dataset with those from the complete-case analysis. In both datasets, the estimated associations between exposure of particulate matters and stroke risk remained consistent, and the difference in effect estimates was not statistically significant (*P* > 0.05). Second, to explore whether the timing of exposure affected our conclusions, we replaced the primary lag structure with alternative 1- and 2-year lags. Again, the revised effect estimates were broadly similar to those of the main models, with no significant difference in parameter estimates (*P* > 0.05). Finally, physical activity was reclassified into three categories (low, moderate, and high) rather than a binary distinction; the results were comparable across the different categorizations, with no statistically significant variation in the main effect sizes (*P* > 0.05). The results of the sensitivity analyses above all confirmed the robustness of the main findings.

## Discussion

Our prospective cohort study of middle-aged and older Chinese adults indicated that higher exposure to PM1, PM_2.5_, and PM10 was significantly associated with increased stroke risk, and that these particulate matters showed a linear relationship with stroke likelihood even after adjustments for demographic, socioeconomic, and health-related variables. Subgroup analyses revealed that these associations were particularly prominent among physically inactive individuals, females, and those who were unemployed or engaged in agricultural work, reinforcing the idea that certain vulnerable populations may need focused intervention. Moreover, the mediation modeling showed that physical activity accounted for part of the relationship between PM_2.5_ exposure and stroke risk, suggesting that reduced physical activity levels could be a significant pathway by which this pollutant exerts its negative impact on cerebrovascular health.

Furthermore, our results are consistent with prior research reporting that particulate air pollutants play a key role in stroke occurrence and progression (21–23). Several large-scale epidemiological studies and meta-analyses have demonstrated that PM_2.5_ exposure, in particular, can significantly increase the risk of acute stroke events, and that its impact may be exacerbated in older adults who often have underlying comorbidities (23, 24). The alignment of our findings with those in other Chinese and international cohorts underscores the importance of stringent control measures for both fine and ultrafine particulate pollution in regions undergoing rapid industrialization. Additionally, our finding that PM_1_ was also consistently and significantly associated with stroke risk adds a novel perspective to the existing literature, which has traditionally focused on PM_2.5_ and PM_10_. Ultrafine particles around or below 1 μm in diameter may penetrate biological barriers more readily, trigger systemic inflammation and oxidative stress, and thus pose an underrecognized threat to cerebrovascular health (25, 26). Given China's ongoing urbanization and rising population of older adults, addressing these pollution-driven risks becomes a critical component of national health policies (27).

In the subgroup analyses, we noted that individuals who were physically inactive, female, or held lower employment statuses exhibited stronger associations between particulate matter and stroke risk. Such heightened susceptibility could stem from multiple factors. Physically inactive individuals may have diminished cardiopulmonary reserve, making them less able to cope with pollution-induced inflammatory or oxidative stress responses (28, 29). Older women often experience hormonal changes, particularly post-menopause, that could exacerbate vascular dysfunction, while employment type may be indicative of socioeconomic constraints, working conditions, and ambient exposures that amplify pollution-related health risks (23, 26). Agricultural workers, for instance, frequently spend prolonged periods outdoors, increasing their exposure to airborne pollutants, whereas unemployed individuals might have fewer resources for protective measures such as air filtration devices or regular clinical check-ups (4, 6, 30).

Moreover, our mediation analysis indicated that physical activity accounted for ~19.6% of the detrimental effect of PM_2.5_ on stroke risk, suggesting that higher pollution levels may discourage active lifestyles, thereby driving up stroke incidence. By contrast, we did not observe such a mediation effect for PM1 or PM10, which may reflect different biological processes or exposure contexts. Specifically, the distinct mediation patterns for PM_1_, PM_2.5_, and PM_10_ could be partly attributable to their varying aerodynamic diameters, chemical compositions, and capacities for alveolar deposition (31). For instance, PM_1_ can penetrate the alveolar-capillary barrier more readily and access systemic circulation, potentially causing localized endothelial damage and microvascular dysfunction that may not heavily depend on physical activity levels for stroke pathogenesis (32). PM_10_, in contrast, tends to deposit in the upper airways and larger conducting airways, triggering inflammatory responses that may not be as closely tied to sedentary behavior. PM_2.5_, given its intermediate size and substantial capacity to reach the pulmonary alveoli while also remaining airborne for extended periods, could uniquely interact with lifestyle factors to influence stroke risk (32, 33). These different mechanistic pathways underscore why only PM_2.5_ showed a significant mediation effect through reduced physical activity, while PM_1_ and PM_10_ might influence stroke via alternative biological or exposure-related mechanisms. Simultaneously, identifying physical activity as a modifiable mediator underscores the potential of targeted interventions, such as safer indoor exercise programs or community facilities with improved air filtration systems, to help mitigate pollution-related health risks in areas with persistently poor air quality (34–36). This is especially relevant for older adults who can benefit substantially from regular physical activity in terms of maintaining cardiovascular health and functional independence (24).

From a mechanistic standpoint, fine and ultrafine particles can penetrate deep into the respiratory tract and enter the bloodstream, triggering systemic inflammation, oxidative stress, and endothelial dysfunction—core processes in the pathophysiology of atherosclerosis and cerebrovascular disease (6, 7, 32, 37). Chronic exposure may heighten coagulation factors and blood viscosity, fostering a pro-thrombotic environment that can precipitate ischemic stroke events (38). Concurrently, individuals with compromised activity levels may exhibit elevated baseline inflammation, abdominal adiposity, and impaired metabolic profiles, further amplifying the atherogenic potential of air pollution (39). Such synergistic effects point to the importance of integrated prevention strategies that address both environmental and lifestyle factors, potentially reducing stroke morbidity and mortality at a population scale (40, 41).

Despite notable strengths such as a large, nationally representative sample, a longitudinal study design, and mixed-effect modeling to account for intra-individual variability over time, this research is subject to certain limitations (42–44). First, the reliance on self-reported stroke status may have introduced classification errors, although validation work in comparable Chinese cohorts has demonstrated acceptable accuracy (42, 45). Moreover, the CHARLS database does not distinguish between ischemic and hemorrhagic strokes, preventing us from exploring whether specific stroke subtypes respond differently to particulate matter exposure. While this represents a key limitation, it does not diminish the overall value of our findings, which highlight the broader link between pollution and cerebrovascular risk. Additionally, although our mediation analysis suggests that physical activity may partially explain the relationship between PM_2.5_ and stroke, physical activity and PM exposures were assessed concurrently, raising potential concerns about temporal ambiguity. Finally, we were unable to control for certain potential confounders, such as detailed dietary patterns or medication use, because these factors were not comprehensively collected in CHARLS. As with all observational studies, residual confounding by unmeasured variables cannot be fully excluded. Future expansions of the survey or additional data linkages could address these gaps, enabling more in-depth analyses of how pollution and lifestyle factors jointly influence stroke risk (46, 47).

In terms of practical translation, our findings underscore the need for multi-level policy measures and community interventions that prioritize resource-limited populations who are most at risk. Examples include providing subsidies or financial support for indoor air filtration devices, particularly in highly polluted areas (39); creating accessible indoor public spaces where individuals can exercise in cleaner air; and offering targeted educational programs to raise awareness about pollution avoidance strategies (31, 32). In addition, tailoring local healthcare services to better reach agricultural workers and unemployed individuals, through mobile health clinics or targeted screening programs, could help diagnose and manage vascular risk factors early. Such approaches can be integrated into broader public health agendas to not only curb the harmful effects of PM pollution but also promote safer physical activity, thereby reducing stroke incidence among under-resourced groups. Lastly, future work incorporating high-resolution exposure metrics and biomarkers of oxidative stress and inflammation would strengthen causal interpretations (48, 49). Meanwhile, developing public health guidelines that encourage safe physical activity, especially for older adults in high-pollution regions, may serve as a practical measure to minimize the burden of stroke (50).

## Conclusion

Our findings highlight that elevated exposure to PM_1_, PM_2.5_, and PM_10_ substantially raises the risk of stroke among middle-aged and older adults in China, with physical inactivity, female sex, and lower socioeconomic status emerging as particularly vulnerable subgroups. These results further indicate that physical activity mediates part of the detrimental impact of PM_2.5_, suggesting the importance of both reducing particulate matter levels through public health and environmental interventions and promoting active lifestyles to mitigate cerebrovascular risks. Future investigations with more precise exposure assessments and biomarkers of inflammation and oxidative stress will help strengthen causal inferences and guide the development of targeted strategies to lessen the burden of pollution-related stroke in aging populations.

## Data Availability

The original contributions presented in the study are publicly available. This data can be found at: https://charls.pku.edu.cn/en/.
